# Neonatal Hypernatremic Dehydration Associated with Lactation Failure

**DOI:** 10.1155/2020/8879945

**Published:** 2020-11-15

**Authors:** Zemichael Ogbe, Amanuel Kidane Andegiorgish, Aradom Habteab Zeray, Lingxia Zeng

**Affiliations:** ^1^Department of Neonatology, Orotta School of Medicine and Health Sciences, Orotta National Referral Hospital, Asmara, Eritrea; ^2^Department of Epidemiology and Biostatistics, Asmara College of Health Sciences, School of Public Health, P.O. Box 8566, Asmara, Eritrea; ^3^Department of Epidemiology and Biostatistics, School of Public Health, Health Science Center, Xi'an Jiaotong University, Shaanxi 710061, China

## Abstract

Hypernatremic dehydration secondary to lactation failure remains a potentially life-threatening condition in countries where advanced laboratory investigations are scarce. An 11-day term baby with excessive weight loss (33.6%), reduced urine output, fever, jaundice, doughy skin, opisthotonus posturing, and tachycardia with poor perfusion was presented to our neonatal care. The baby was diagnosed with shock with hypernatremic dehydration. An initial bolus of 20 ml/kg of N/S was repeated 3 times (each over 20 minutes), i.e., a total of 204 ml was given over 1 hr, until the vital signs were normalized to PR-145, RR-45, T-37.2°C, SPO_2_-100%, and CRT < 3 seconds, and the baby began to void urine. Free water deficit and sodium excess was managed by gradual and slow correction over 72 hours to prevent cerebral oedema and neurologic sequelae. The baby required reconstituted solutions of 5% D/W + 1/2 N/S at a rate of 27 ml/hr for 72 hrs. Sepsis and hyperbilirubinemia were treated with antibiotics and phototherapy. Management of symptomatic hypernatremic dehydration must be considered in settings with inadequate laboratory facilities.

## 1. Introduction

Hypernatremic dehydration (HND), defined as serum sodium concentration > 145 mmol/L [mEq/L] [1, 2], is a potential lethal condition for neonates. Dehydration in newborns presents in three forms: hyponatremic, normonatremic, and hypernatremic depending on the serum osmolality. This hyperosmolar state if not treated immediately can lead to brain shrinkage, subdural capillary hemorrhage, venous thrombosis, gangrene, and death [3–5]. Even though, several studies have documented the causes of HND are either decreased fluid intake, excessive fluid loss, or excessive sodium intake [1, 2, 6, 7]; a study by Oddie et al. has concluded that the sole cause of HND is unsuccessful breastfeeding (lactation failure or sometimes referred as “Breast-Feeding Malnutrition”) [8, 9]. Besides the capacity of ongoing milk synthesis (galactopoiesis), adequate breast milk intake depends on effective milk removal, which includes breastfeeding techniques, combined with total daily milk intake on frequency and duration of feeds [10]. Early discharge from health facilities with inadequate training on proper breastfeeding practice is increasingly documented as major causes of HND [11, 12]. Hence, the benefit of 1^st^ week of life baby and mother monitoring for successful breastfeeding has become more prominent [2].

It is normal for neonates to lose as much as 7% of their birth weight on the first week of their life through normal dieresis. Thereafter, neonates should start to regain their weight by the 10^th^ day of their life [13].

The first sign of neonatal hypernatremic dehydrations is weight loss (>7% of birth weight) combined with failure to have bowel movement or the presence of urate crystals [13–15]. However, pediatricians and attending physicians in low-income countries with inadequate facility for early diagnosis are facing difficulties at management of this uncommon but easily preventable complication.

## 2. Case Report

**Figure 1:**
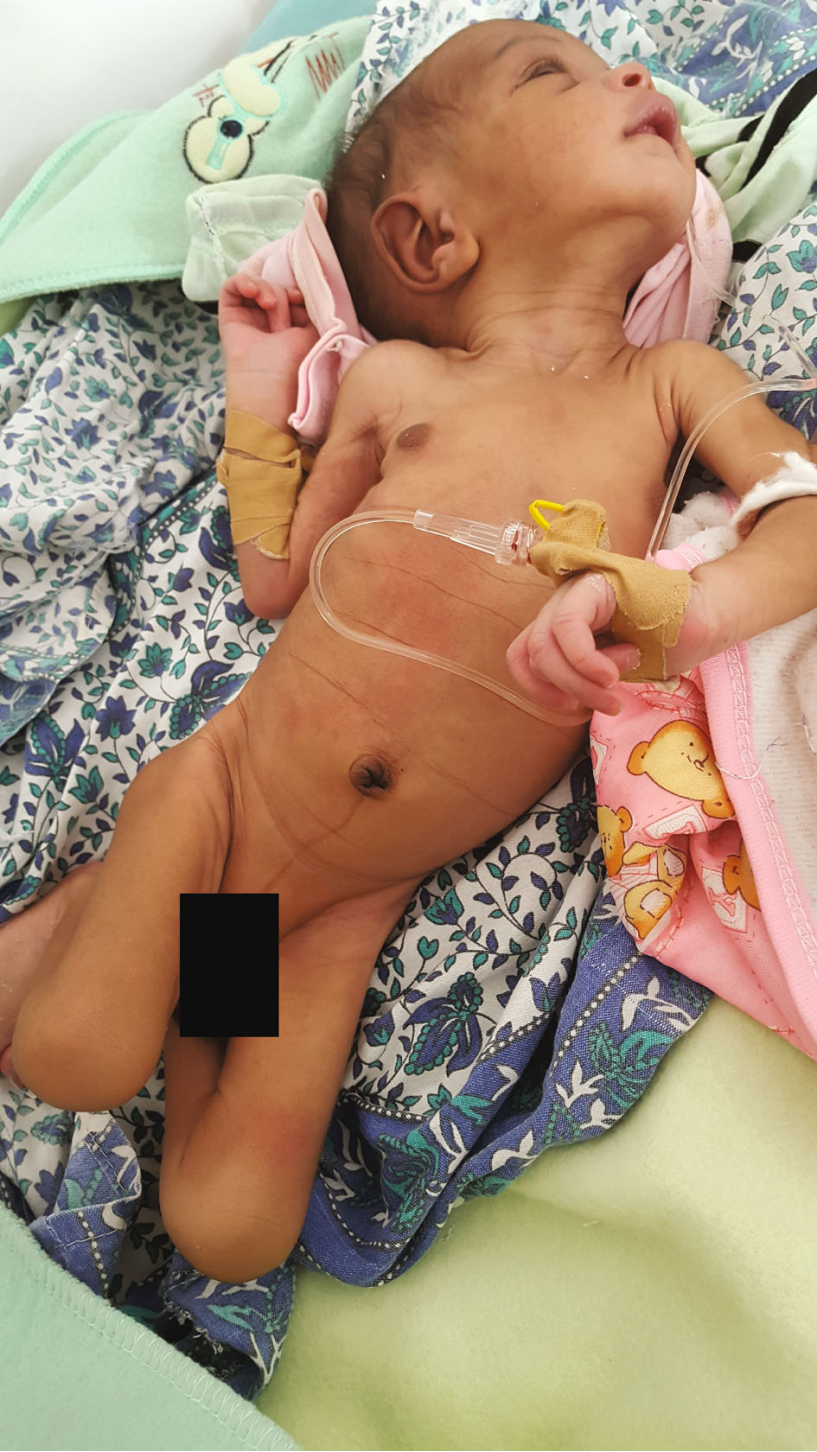
Picture of a 12 -day neonate on admission with hyperextended neck.

An 11-day-old male baby was admitted to the neonatal ward (W/A) (Specialized Neonatal Care Unit) in Orotta National Referral Hospital, Paediatric Department, in Asmara, the capital city of Eritrea on March 8, 2018, at 9 : 20 pm (Figure 1).

The baby was a product of a term gestation, 39 weeks and 6 days by date, born to a 29-year-old multiparity mother whose pregnancy was uneventful, and having previous positive feeding experience. Delivery was via normal spontaneous vaginal with a birth weight of 3.4 kg at a health station in a village called Beleza, about 12 km from the capital city Asmara and was discharged home after 6 hrs of delivery.

On admission, he was in a moribund state with a history of poor sucking for 4 days, absent urine-output for 2 days, fever for the last 2 days, and hyperextended neck and extremities for 1 day. According to the mother, the baby was doing fine soon after birth and before the onset of the illness.

The mother reported that the baby was exclusively breastfed 5-6 times/24 hrs, and she considered him to be a quiet baby who was easily satisfied.

By 6-7 days of age, the mother noticed that he was not well as before. He started to experience irritability, poor sucking, and reduced urination that eventually got worse until the mother sought medical attention. There had been no vomiting and diarrhea.

Physical examination revealed that a sick baby weighed 2.26 kg. Vital signs were PR-175 p/m, RR-irregular 38 bp/m, T-38.3°C, SPO_2_-20%, and cold extremities with capillary refill time (CRT) > 3seconds. The baby was lethargic with opisthotonus posturing, labored deep breathing with marked chest retractions, icteric sclera, dry lips, and doughy abdominal skin. Findings during the remainder of the physical examination were unremarkable.

Laboratory investigations: on day of admission were limited only to the following:

- CBC-, WBC-17.7, RBC-5.71, HGB-20.8, HCT-62.9, PLT-40
- RBS-51 mg/dl
- Total serum Bil.–19.5 mg/dl

With the abovementioned clinical manifestations, the baby was diagnosed as HND with shock as the major diagnosis.

The baby was treated with the initial boluses of 20 ml/kg of N/S (Table 1), repeated 3 times (each over 20 minutes), that is, a total of 204 ml was given over 1 hr, until the vital signs were normalized to PR-145, RR-45, T-37.2°C, SPO_2_-100%, and CRT < 3 seconds, and the baby began to void urine. Then, free water deficit and sodium excess were managed by gradual and slow correction over 72 hours to prevent cerebral oedema and neurologic sequelae. The baby required reconstituted solutions of 5% D/W + 1/2 N/S at a rate of 27 ml/hr for 72 hrs.

In view of the sick appearance, fever, and low platelet count, sepsis was considered, and therefore, antibiotics were administered. Hyperbilirubinemia was reduced to normal with one-day exposure to phototherapy.

By day 3, the serum sodium level was 158 mmol/L so the fluid was shifted to 10% D/W + 1/4 N/S.

The serum sodium levels returned to normal by day 5 of admission (Table 2). Blood urea nitrogen and creatinine reached the normal range by day 7 of admission, and their values were 16 mg/dL and 0.5 mg/dL, respectively. The baby started to feed completely orally by day 4 of admission. Daily weight monitoring indicated that the baby started to gain rapidly and gained his original birth weight by the 6^th^ day after he started feeding, i.e., by the 10^th^ day after admission; then, the rate of increment slowed down to reach 3.9 kg on discharge.

The baby was discharged on day 14 after admission, one week after complete stabilization without any complication; during that time, extensive training on breastfeeding was conducted and proper breastfeeding was ensured.

Babies with severe HND in our setup are usually referred to a high-risk clinic for further follow-up to detect early neurological sequelae after discharge from the hospital. Monthly follow-up revealed that there was no evidence of neurological sequelae nor was any delay in developmental milestones in the first year and 2-3 months follow-up in the second year of life.

## 3. Discussion

Neonatal hypernatremic dehydration is a medical emergency with high rate of morbidity and mortality. Early diagnosis of HND among exclusive breast milk feeding neonates is crucial for prompt treatment and survival of the baby, but diagnosis is often difficult and remained unnoticed by mothers and attending physicians.

Probably, this is why HND associated with breastfeeding failure had inadequate exposure in medical literature especially from low income countries. Of the several reasons, one could be HND was considered to occur in those babies who were fed with artificial feeds, powdered milk with high sodium concentration, especially when the mother failed to add enough water in the mixture [8]. The second plausible speculative explanation for this is that HND might have been unrecognized or misdiagnosed, as associated clinical manifestations in most cases resemble sepsis or are associated with sepsis, and treatment of sepsis was taken as major management for this condition [16]. Another important clinical manifestation associated with HND is jaundice [17], and because hypernatremia can cause impairment in the blood-brain barrier function, which may enhance the diffusion of bilirubin across the blood-brain barrier and thereby may enhance the risk of bilirubin encephalopathy [18].

In this case report, the opisthotonos posturing of the baby might have been attributed by hyperbilirubinemia, but after fluid resuscitation and phototherapy, the patient recovered without any neurological sequelae, as confirmed in subsequent follow-up.

HND due to breastfeeding failure is attributed to low breast milk production (delayed breast milk maturation) and inadequate breastfeeding, which is related to high concentration of sodium in breast milk [6]. Normally, matured breast milk is low in sodium concentration, and this protects against the development of HND in exclusively breast-fed newborns. Studies of the electrolyte composition of breast milk have shown a mean sodium value of 64.8 ± 4.4mmol/L after delivery, dropping to a mean of 21.4 ± 2.3mmol/L by the third postpartum day (colostrum), and leveling off to a value of 7 ± 2mmol/L by 2 weeks (mature milk) [19]. Therefore, variation in the normal physiology and maintaining the high levels of sodium concentration in breast milk are closely associated with lactation failure [7, 20].

Clinical presentations of hypernatremic dehydration due to lactation failure usually are around ten days with the range from 3^rd^ to 21^st^ days of life [21] consistent to the present case. Sleepy and quiet (not appearing hungry) baby presentations make early recognition of HND extremely difficult and water shift from the intracellular to the extracellular compartment keeping normal skin turgor mislead the underlying dehydration. Delayed noting deteriorates the condition and precipitates the baby's emergency admission.

The patient in this case report presented in severe form of HND with excessive weight loss, reduced urine output, fever, jaundice, doughy skin, opisthotonus posturing, and tachycardia with poor perfusion which required emergency resuscitation with boluses of fluid. After that, further management should depend on monitoring of serum sodium level and clinical manifestations to normalize the serum sodium level [22].

The demographic characteristics: multiparity, male baby, and greater weight loss were similar to features of neonates associated for neonatal HND [23]. Unfortunately, our laboratory is not always in a position to provide the available investigations at any time. The diagnosis of HND on admission was on a clinical basis as the baby was admitted in the evening, and the necessary laboratory investigations were not available. Renal function test and electrolytes were done only on a daily basis.

In this case report, the second aim is to state the difficulties pediatricians and the attending physicians may face at the management of HND.

In a setup where there are limited laboratory investigations, the diagnosis of HND should be highly considered, if the baby presents with poor sucking, excessive weight loss more than 7% of the birth weight, and reduced frequency or absent urine output which are sensitive indicators of HND [2].

HND should not be corrected rapidly because of the associated cerebral oedema. The goal is to decrease the serum sodium concentration by 12 mmol/L/24 hrs for over 2-4 days [24]. This needs frequent, 4-6 hourly monitoring of serum sodium concentration to adjust the sodium concentration of the deficit replacement. This was not applicable in this case report as the monitoring of sodium was on a daily basis. We used the general recommendations for treatment of HND consist of an emergency phase (restoration of vascular volume with 20 ml/kg of N/S) followed by rehydration phase (the sum of free water deficit and maintenance fluid requirement administered evenly over 48-72 hrs [25] as shown in Table 1.

More often, we encountered babies with HND whose birth weights are not known, especially those who were delivered at home, calculating the fluid deficit can be estimated by the degree of dehydration. Besides, the clinical manifestation in HND is variable and sometimes misleading. In this case, multiplying the fluid maintenance by 1.5 will be the best approximation to correct the water deficit and sodium excess with 5% D/W + 1/2 N/S during the rehydration phase: [(presentWt.×maintenancefluid/kg × 1.5)minusthebolusesgivenduringemergencyphase] [24].

Recognizing lactation failure early can be difficult not only for parents but for health personnel also. In this case report, although the mother is multipara and has experience of breastfeeding the previous siblings, she frankly admitted that she was breastfeeding 5-6 times per day only, and it is difficult to quantify the amount and the duration at each breastfeeding given. On top of this, we believe that sepsis and jaundice had contributed to the severity of the lactation failure in this particular patient. In a setup with ill-prepared facilities, a baby with fever, jaundice, and low platelets on minimum laboratory investigations, infection should be considered and antibiotics should be started immediately. Starting antibiotics to save the baby's life is a priority rather than waiting to confirm the laboratory investigation. This is supported by the WHO recommendation for poorly resourced countries [26].

Generally, mothers in Eritrea traditionally breastfeed exclusively because it is believed to be the most appropriate source of nutrition for the very young infants, but what is missing for them is training and adequate technical skills on breastfeeding.

## 4. Conclusion

HND is a serious, life-threatening disorder with high rates of mortality and morbidity. Early diagnosis and prompt management of HND due to lactation failure by identifying simple clinical predictors is critically important to prevent deaths of neonates, especially in resource-limited countries. Therefore, physicians, midwives, community nurses, and nurse assistants should be equipped with knowledge to early identify newborns at risk for HND and prevent it by body weight monitoring of baby for the first 3 weeks of life, until a normal weight velocity is recorded as recommended by the American Academy of Pediatrics, to allow early identification of HND [8] and providing adequate instructions on breastfeeding to the mothers [3].

In countries with limited resources, to improve parental knowledge on breastfeeding and monitor the inadequate fluid intake that leads to HND, the health personnel at any level of health facility should educate mothers about adequate breast-feeding methods, to search for the early signs of dehydration in infants and providing educational brochure upon discharge. We recommend a modified Korean educational brochure for mothers [27] (Table 3).

## Figures and Tables

**Table 1:** Calculation used to correct the fluid deficit.

General information
Birth weight = 3.4 kg, admission weight = 2.26 kg
Weight loss = 1.14 kg, percent dehydration = 1.14/3.4 = 33.6%
Free water loss = 1140 ml
Fluid therapy
Step 1: emergency phase
Restore vascular volume with boluses 20 ml/kg of N/S
20ml × 3.4kg = 68ml over 20 minutes repeated 3 times = 204 ml.
Step 2: rehydration phase
Aim to correct water deficit and sodium excess within 72 hours.
□ Maintenance during 3 days: 100ml/kg/d × 3.4kg × 3days = 1020ml
□ Maintenance deficit = 1020ml + 1140ml = 2160ml, and take initial hydration fluid out of this.
2160ml − 204ml = 1956ml
□ 956ml/72hr≒27ml/hr of 5% dextrose + 1/2 normal saline.

**Table 2:** Subsequent laboratory investigations on a daily basis disclosed the following values (mmol/L).

	ALB	BUN	Cr	Tot. Bil	Na	K	Cl	Ca
Day 1		238	8.2	19.5	180	9.6	148	6.8
Day 2	2	211	6	15.4	168	7.1	129	9.6
Day 3	2.5	189	5.2	13.0	158	5.6	129	7.2
Day 4	2.5	168	4.2	11	150	5.3	121	7.8
Day 5	2.8	148	3.5	9	145	5.5	120	8,0
Day 6	3.0	114	2.1	6.0	143	5,4	117	8.0
Day 7	3.5	16	0.5	5.1	142	5.0	105	9.0

**Table 3:** Modified Korean educational brochure for mothers.

Signs that your baby is breast-feeding well
(i) Has wet diaper at least 4-5 times in 24 hrs and the baby looks well (ii) Has at least 2-3 bowel movements in 24 hrs (iii) Breastfeed at least 8 times in 24 hrs (iv) The baby is content after feeding (v) You can hear your baby swallowing during breastfeeding (vi) Mother's breasts are full before feeding and soft after feeding (vii) Your baby is only drinking breast milk
If at least one of the above signs is absent, then the mother should visit the nearby health facility immediately for evaluation, proper management, or counseling.

## References

[1] Späth C., Sjöström E. S., Ahlsson F., Ågren J., Domellöf M. (2017). Sodium supply influences plasma sodium concentration and the risks of hyper- and hyponatremia in extremely preterm infants. *Pediatric Research*.

[2] Mujawar N., Jaiswal A. (2017). Hypernatremia in the neonate: neonatal hypernatremia and hypernatremic dehydration in neonates receiving exclusive breastfeeding. *Indian Journal of Critical Care Medicine*.

[3] Kaplan J. A., Siegler R. W., Schmunk G. A. (1998). Fatal hypernatremic dehydration in exclusively breast-fed newborn infants due to maternal lactation failure. *The American Journal of Forensic Medicine and Pathology*.

[4] Moritz M. L., Ayus J. C. (2005). Preventing neurological complications from dysnatremias in children. *Pediatric Nephrology*.

[5] Dogra S., Agrawal S. K., Jindal R., Suri D., Ahluwalia J., Singh S. (2011). Peripheral gangrene in a breast fed neonate--is hypernatremic dehydration the cause?. *Indian Journal of Pediatrics*.

[6] Peters J. M. (1989). Hypernatremia in breast-fed infants due to elevated breast milk sodium. *The Journal of the American Osteopathic Association*.

[7] Bhave G., Neilson E. G. (2011). Body fluid dynamics: back to the future. *Journal of the American Society of Nephrology*.

[8] Oddie S., Richmond S., Coulthard M. (2001). Hypernatraemic dehydration and breast feeding: a population study. *Archives of Disease in Childhood*.

[9] Koklu E., Gunes T., Ozturk M. A., Kose M., Kurtoglu S., Yuksel F. (2007). A review of 116 cases of breastfeeding-associated hypernatremia in rural area of central Turkey. *Journal of Tropical Pediatrics*.

[10] Koo W. W., Gupta J. M. (1982). Breast milk sodium. *Archives of Disease in Childhood*.

[11] Boskabadi H., Maamouri G., Ebrahimi M. (2010). Neonatal hypernatremia and dehydration in infants receiving inadequate breastfeeding. *Asia Pacific Journal of Clinical Nutrition*.

[12] Bolat F., Oflaz M. B., Güven A. S. (2013). What is the safe approach for neonatal hypernatremic dehydration?. *Pediatric Emergency Care*.

[13] Section on Breastfeeding (2005). Breastfeeding and the use of human milk. *Pediatrics*.

[14] Staub E., Wilkins B. (2012). A fatal case of hypernatraemic dehydration in a neonate. *Journal of Paediatrics and Child Health*.

[15] Livingstone V. H. (1990). Problem-solving formula for failure to thrive in breast-fed infants. *Canadian Family Physician Medecin de famille canadien*.

[16] Yildiz N., Erguven M., Yildiz M., Ozdogan T., Turhan P.℩. (2013). Acute peritoneal dialysis in neonates with acute kidney injury and hypernatremic dehydration. *Peritoneal Dialysis International*.

[17] Lavagno C., Camozzi P., Renzi S. (2016). Breastfeeding-associated hypernatremia. *Journal of Human Lactation*.

[18] Wennberg R. P., Johansson B. B., Folbergrova J., Siesjo B. K. (1991). Bilirubin-induced changes in brain energy metabolism after osmotic opening of the blood-brain barrier. *Pediatric Research*.

[19] Heldrich F. J., Shaw S. S. (1990). Case report and review of literature: hypernatremia in breast-fed infants. *Maryland Medical Journal*.

[20] Humenick S. S., Hill P. D., Thompson J., Hart A. M. (1998). Breast-milk sodium as a predictor of breastfeeding patterns. The Canadian journal of nursing research. *Revue canadienne de recherche en sciences infirmieres*.

[21] Das J. (2015). Hypernatremic dehydration in newborn infants: a review. *The Ulutas Medical Journal*.

[22] Schwaderer A. L., Schwartz G. J. (2005). Treating Hypernatremic Dehydration. *Pediatrics in Review*.

[23] Ferrández-González M., Bosch-Giménez V., López-Lozano J., Moreno-López N., Palazón-Bru A., Cortés-Castell E. (2019). Weight loss thresholds to detect early hypernatremia in newborns. *Jornal de Pediatria*.

[24] Marcdante K., Kliegman R. M. (2015). *Nelson Essentials of pediatrics*.

[25] Rand S. E., Kolberg A. (2001). Neonatal hypernatremic dehydration secondary to lactation failure. *The Journal of the American Board of Family Practice*.

[26] (2018). *WHO Sepsis Technical Expert Meeting - Meeting report*.

[27] Oh Yun J., Lee Ji E., An So H. (2007). Severe hypernatremic dehydration in a breast-fed neonate. *Korean Journal of Pediatrics*.

